# Depression and unplanned secondary healthcare use in patients with multimorbidity: A systematic review

**DOI:** 10.1371/journal.pone.0266605

**Published:** 2022-04-07

**Authors:** Meryem Cicek, Benedict Hayhoe, Michaela Otis, Dasha Nicholls, Azeem Majeed, Geva Greenfield

**Affiliations:** 1 Applied Research Collaboration Northwest London (ARC NWL), School of Public Health, Faculty of Medicine, Imperial College London, London, United Kingdom; 2 Division of Psychiatry, Department of Brain Sciences, Faculty of Medicine, Imperial College London, London, United Kingdom; Faculty of Health Sciences - Universidade da Beira Interior, PORTUGAL

## Abstract

**Background:**

Growing numbers of people with multimorbidity have a co-occurring mental health condition such as depression. Co-occurring depression is associated with poor patient outcomes and increased healthcare costs including unplanned use of secondary healthcare which may be avoidable.

**Aim:**

To summarise the current evidence on the association between depression and unplanned secondary healthcare use among patients with multimorbidity.

**Methods:**

We conducted a systematic review by searching MEDLINE, EMBASE, PsychINFO, Web of Science, CINAHL, and Cochrane Library from January 2000 to March 2021. We included studies on adults with depression and at least one other physical long-term condition that examined risk of emergency hospital admissions as a primary outcome, alongside emergency department visits or emergency readmissions. Studies were assessed for risk of bias using The National Institute of Health National Heart, Lung, and Blood Institute quality assessment tool. Relevant data were extracted from studies and a narrative synthesis of findings produced.

**Results:**

Twenty observational studies were included in the review. Depression was significantly associated with different outcomes of unplanned secondary healthcare use, across various comorbidities. Among the studies examining these outcomes, depression predicted emergency department visits in 7 out of 9 studies; emergency hospital admissions in 19 out of 20 studies; and emergency readmissions in 4 out of 4 studies. This effect increased with greater severity of depression. Other predictors of unplanned secondary care reported include increased age, being female, and presence of greater numbers of comorbidities.

**Conclusion:**

Depression predicted increased risk of unplanned secondary healthcare use in individuals with multimorbidity. The literature indicates a research gap in identifying and understanding the impact of complex multimorbidity combinations, and other patient characteristics on unplanned care in patients with depression. Findings indicate the need to improve planned care for patients with moderate-to-severe depression. We suggest regular reviews of care plans, depression severity monitoring and assessment of hospital admission risk in primary care settings.

## Introduction

Multimorbidity, the co-occurrence of two or more chronic conditions [1], is increasing in prevalence and affecting approximately a third of all adults globally [2]. In the UK, the prevalence of individuals with four or more long-term conditions is projected to increase to 17% by 2035, compared to 9.8% in 2015 [3]. Approximately two thirds of this population will have a mental illness such as depression [3], which is in turn strongly associated with the incidence of a multitude of long-term conditions [4–6]. As the number of physical conditions a person increases, the odds of having a mental health disorder increase by almost double for one condition, and six times for more than five conditions [7]. The presence of a mental health comorbidity such as depression is associated with poorer clinical outcomes and quality of life, compared to individuals with physical conditions only [8–10].

Individuals with multimorbidity have complex needs requiring long-term management and treatment of multiple conditions across multiple healthcare settings. Multimorbidity, in general, is associated with increased health service utilisation across primary care, planned secondary care, urgent care and dental care [11–13] and with increased costs related to medications and care transitions [12]. From a broad range of chronic conditions, patients with depression alongside physical comorbidities produce the greatest cost-increasing effect in primary care costs [14]. The impact of multimorbidity on available resources varies based on differences across healthcare systems, comorbidity combinations and patient factors such as frailty, social care access, and socioeconomic deprivation [12, 14, 15]. This is a significant challenge for healthcare systems in planning and delivering services for patients with multimorbidity.

Specifically, the use of unplanned secondary healthcare may be considered as a proxy for inadequate or unsuitable planned care for individuals with multimorbidity [16, 17] and, importantly, is potentially preventable. Excess unplanned care is more expensive for patients and providers than routine care thus earlier prevention can reduce related costs [18]. Some studies have investigated the effect of having any type of mental health condition on general secondary care usage [19, 20] while others focus on depression [17, 21]. A few studies indicate that depression is associated with various types of unscheduled healthcare utilisation such as urgent general practitioner visits, hospital admissions and emergency department (ED) visits in people with one other chronic condition [21, 22].

A previous systematic review and meta-analysis [22] conducted in 2012 looked at the association between depression and any type of urgent healthcare in patients with either asthma, chronic obstructive pulmonary disorder (COPD), coronary heart disease (CHD) or diabetes. An updated review is needed to include new literature that also adopts a broader scope to multimorbidity beyond a limited number of pre-determined conditions.

The aim of this review is to summarise the current evidence on the association between depression and unplanned secondary healthcare use among patients with multimorbidity. To our knowledge, the literature is limited on specifically depression-related multimorbidity clusters, namely different combinations of comorbidities, or specific patient characteristics and the subsequent effect on unplanned secondary healthcare use. Therefore, this review also aims to explore the effect of the types of comorbidities and if available, different clusters of comorbidities, and sociodemographic predictors of unplanned secondary healthcare among patients with both multimorbidity and depression.

## Methods

A review of the current literature was conducted in accordance with the Preferred Reporting Items for Systematic Reviews and Meta-Analysis (PRISMA) guidelines (S1 Table). An initial protocol was registered on PROSPERO (CRD 42021237356).

### Eligibility criteria

Studies were eligible if they included adults aged 18+ with at least one other long-term condition in addition to depression and considered emergency hospital admissions as one of their main outcomes. Observational studies and randomised controlled studies comparing individuals with and without depression were considered for inclusion. Only studies using a standardised and validated measure of identifying depression in patients and reporting quantified measures of association such as risk ratios, odds ratios, or hazard ratios were included. Studies were excluded if participants were already in an emergency department or a current inpatient at the time of recruitment to the study. This is because the focus of our outcome is to determine the risk of an individual coming in to use the emergency department, including those with readmission risk, not for patients already found there. No filter restrictions were applied except for publication date.

### Information sources and search

Searches in Medline, Embase, PsycINFO, Web of Science, CINAHL and Cochrane Library were performed in March 2021, for records since January 2000. Reference lists of relevant studies were hand-searched for further relevant publications. A combination of search terms was used to identify relevant articles, such as “multiple long-term condition/comorbid”, “depression”, and “emergency care”. The search strategy was developed with the assistance of a specialist librarian at Imperial College London. Search terms and syntax were adapted for each individual database (S2 Table).

### Screening and selection

Records captured through electronic database searching and hand-searching were de-duplicated. Two reviewers (MC and MO) independently screened the titles and abstracts of the remaining records for inclusion based on eligibility criteria. The same two reviewers independently reviewed the full text of eligible articles. Discrepancies were resolved through discussion with the other authors.

### Data extraction

A data extraction form was developed and used to capture key data from records relevant to the review aims. Data extracted included study characteristics, sociodemographic and clinical characteristics associated with emergency hospital admissions and emergency department visits as per the study findings, and measure of risk of unplanned secondary healthcare use in respective study populations. Primary data extraction was performed by MC and was reviewed by MO.

### Risk of bias assessment

Following full-text screening of relevant articles, two reviewers (MC & MO) independently appraised the included studies for the risk of bias by using the National Heart, Lung, and Blood Institute (NHBLI) quality assessment tool for observational and cross-sectional studies [23] (see S3 Table). This tool was suitable as all included studies employed an observational or cross-sectional design. Minor differences were discussed amongst the authors.

### Outcome measures

The main outcome of interest was the risk of emergency hospital admission, and secondary to that emergency department visits and/or emergency hospital readmissions as measures of unplanned secondary healthcare use. The rationale for this is that patients who undergo emergency hospital admission will have likely passed through the emergency department, thus capturing the outcome more likely to indicate severe patient condition.

### Data synthesis and analysis

Evidence tables were produced to include relevant data on the study characteristics, clinical and sociodemographic predictors, and key findings. Certainty of outcome measures (i.e., risk ratio, odds ratio, hazard ratio) reported were assessed prior to synthesis based on 95% confidence intervals and statistical significance threshold of p<0.05. A narrative synthesis of the data was conducted to summarise key findings by grouping studies with similar outcomes, clinical and sociodemographic predictors of unplanned secondary healthcare, as available in included studies. Data collected from the studies were not suitable for meta-analysis due to the heterogeneity of the outcome measures across the studies.

## Results

**Fig 1:**
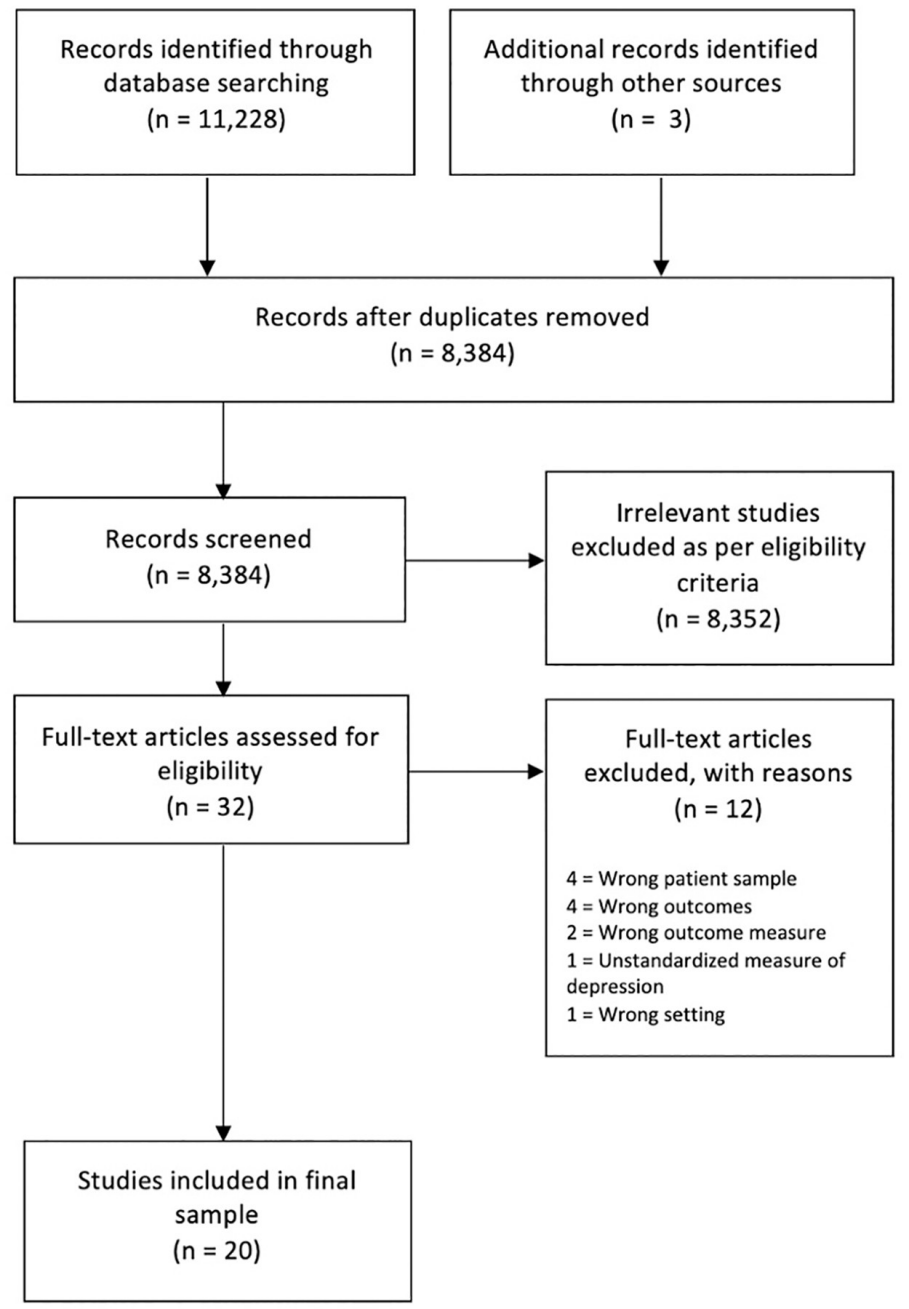
PRISMA inclusion flowchart.

Database searches identified 11,228 records, with three identified through hand-searching. After removing 2,874 duplicates, 8,384 titles and abstracts were screened, of which 8,352 did not meet the eligibility criteria. Full texts were obtained for thirty-two articles of which a further 12 articles were excluded (see Fig 1 for PRISMA chart); 20 studies were included in the final set (Table 1).

**Table 1:** Characteristics and findings of studies included in systematic review.

Author/Date	Year(s)	Country	Study Design	Sample Size	Sample Population	Depression Measure	Index Comorbidity	Key Findings
**Bhatt et al 2016** [24]	2007–2011	USA	Prospective cohort	308	Outpatients enrolled in a prospective HF cohort study	Patient Health Questionnaire-9 (PHQ-9), scores stratified as minimal (0–4), mild (5–9), or moderate-to-severe (10–27).	Heart failure (HF)	Having moderate-to-severe depressive symptoms predicted 1.70 times higher risk of all-cause admissions and 2.5 times more HF-related admissions than patients without depressive symptoms. When adjusted, those with mild depressive symptoms had 1.57 times greater risk of all-cause admissions compared to patients without depression. Depressive symptoms were not associated with ED visits.
**Blakemore et al 2019** [25]	2013–2014	UK	Longitudinal cohort	355	Patients with COPD in six primary care practices in the UK	Hospital Anxiety and Depression Scale (HADS), scores stratified as 4 quartiles	COPD	Subthreshold depression (HADS depression score 4–7) was associated with a 2.8 times increased odds of emergency hospital admission, and HADS depression >8 was associated with 4.8 times increased odds.
**Doubova et al 2018** [26]	2016	Mexico	Cross-sectional	192	T1D patients 18 years+ who attended follow-up visits at the endocrinology department	Medical record diagnosis	Type-1 Diabetes Mellitus	Depression associated with emergency room use (adjusted PR = 1.72, p = 0.049) but not significantly associated with diabetes-related hospitalizations in patients with type 1 diabetes (adjusted PR = 0.85, p = 0.922).
**Eisner et al 2005** [27]	2000–2003	USA	Prospective cohort	743	Adults with asthma who were recruited after hospitalization for asthma	Depressive symptoms defined as having a score of 16+ on the Center for Epidemiologic Studies Depression Scale (CES-D)	Asthma	Depression not associated with ED visits (HR = 1.36, p = 0.12) but predicted hospital admission (HR = 1.34, p = 0.06)
**Ghanei et al 2007** [28]	2006–2007	Iran	Prospective cohort	157	Patients attending chest clinic during 2006	Hospital Anxiety Depression Scale (HADS)	COPD	Depression predicted hospital admission due to COPD exacerbation (RR = 0.31, p = 0.05)
**Guthrie et al 2016** [21]	2010	UK	Prospective cohort	1,860	Primary care patients in socially deprived areas of Manchester	Hospital Anxiety Depression Scale (HADS), scores stratified as 5 quintiles	Diabetes, ischaemic heart disease (IHD), COPD or asthma	Having depression independently associated with an increased risk of prospective emergency admission to hospital (OR 1.58, 95%CI 1.04–2.40). Compared to baseline 0–1 HADS score, statistically significant adjusted OR for prospective emergency admissions in patients with depression was OR = 2.42 (p = 0.025) for HADS score 11+.
**Himelhoch et al 2004** [29]	1999	USA	Cross-sectional	60,382	Adults aged 65+ with Medicare part A and B fee-for-service coverage in 1999	ICD-9 codes for range of depressive syndromes. To be defined as having depression, participants required to have 1+ inpatient/2+ outpatient claims with depressive syndrome code.	Coronary artery disease, diabetes, congestive heart failure, hypertension, prostate cancer, breast cancer, lung cancer, or colon cancer.	For all 8 comorbidities, patients with depression were at least twice as likely to have both emergency department visits and at least three times as likely to have hospital admissions and were all statistically significant even after adjustment.
**Katon et al 2013** [30]	2000–2007	USA	Prospective cohort	4,117	Adults aged 18+ from the Pathways Epidemiologic Follow-up Study cohort, from 9 primary care clinics in Western Washington	Patient Health Questionnaire-9 (PHQ-9)	Type 1 or 2 Diabetes Mellitus	Depression significantly predicted time to first severe hypoglycaemic episode requiring an emergency department visit or hospitalization (aHR = 1.42, 95%CI 1.03–1.96) and number of hypoglycaemic episodes (aOR = 1.34, 95%CI 1.03–1.74), even after adjusting for prior hypoglycaemic event and demographic, clinical, and health risk behaviour characteristics.
**Laurence et al 2017** [31]	2008	USA	Cross-sectional	36,420	Patients aged 50+ diagnosed with head and neck cancer	ICD-9 codes for range of depressive syndromes.	Head and neck cancer (HNC): larynx/ hypopharynx, oropharynx, oral cavity.	Depression was associated with greater likelihood of emergency admissions for overall HNC, slightly higher among women (PR = 1.31, 95%CI 1.20–1.42) compared to men (PR = 1.28, 95%CI 1.21–1.36),
**Laurence et al 2019** [32]	2008	USA	Cross-sectional	113,831	Patients aged 50+ diagnosed with HIV	ICD-9 codes for range of depressive syndromes.	HIV	Depression diagnosis increased the likelihood of emergency hospital admission after adjusting for demographic and hospital characteristics and comorbidities (PR = 1.45, 95% CI 1.39–1.52).
**Mausbach et al 2017** [33]	2011	USA	Retrospective observational	5,055	Outpatients with an ICD-9 diagnosis of any cancer at University of California San Diego Healthcare System	ICD-9 codes for range of depressive syndromes from medical records.	Cancer	Having depression was significantly associated with more ED visits (OR = 2.45; 95% CI 1.97–3.04), overnight hospitalizations (OR = 1.81; 95% CI 1.49–2.20), and 30-day hospital readmission (OR = 2.03; 95% CI 1.48–2.79)
**Moraska et al 2013** [34]	2007–2010	USA	Prospective cohort	402	Patients with heart failure identified from electronic medical records across medical providers in southeast Minnesota	Patient Health Questionnaire (PHQ-9), scores stratified as none-minimal (0–4), mild (5–9), or moderate-severe (10+).	Heart failure	Having moderate-severe depression was associated with an increased risk of hospitalization (HR 1.79, 95% CI 1.30–2.47) and emergency department visits (HR 1.83, 95% CI 1.34–2.50).
**Niazi et al 2018** [35]	1991–2010	USA	Retrospective observational	47,608	Patients with multiple myeloma on National Cancer Institute’s Surveillance, Epidemiology, and End Results (SEER) registry	ICD-9 code for depression.	Multiple myeloma	Compared to those without depression, patients with depression had statistically significant greater odds of emergency department care (OR = 1.37, 95%CI 1.28–1.47) and inpatient hospital admissions (1.41, 95%CI 1.31–1.53), after adjusting for age, calendar year, sex, race, and Charlson comorbidity index.
**Pan et al 2015** [36]	2006–2009	USA	Cross-sectional	4,766	Adult survivors of cancer aged 21+ from the household Medical Expenditure Panel Survey (MEPS)	ICD-9-CM codes for depression.	Cancer	Those with depression had significantly greater likelihood of using emergency departments (aOR = 1.46, 95%CI 1.17–1.82) and compared to those without depression.
**Poojary et al 2017** [37]	2013	USA	Cross-sectional	26,094	Adults aged between 18–80 in the United States National Readmission Database	ICD-9-CM codes for depression.	Ulcerative colitis	Depression predicted unplanned readmissions (aOR 1.40, 95% CI, 1.16–1.66) in patients with a primary UC diagnosis.
**Ricketts et al 2018** [17]	2015–2016	UK	Retrospective observational	469,368	Adults aged 18+ and registered in primary care in city of Sheffield	Depression diagnosis recorded in primary care records.	19 Long-term conditions (recorded in UK Quality Outcomes Framework)	Those with depression and ≥1 LTC had significantly greater likelihood of using unplanned hospital care than individuals with LTC only (aOR = 1.59, p < .001), after adjusting for age, deprivation and no. of conditions.
**Schneider et al 2008** [38]	2005–2006	Germany	Prospective observational	185	Patients with asthma from 46 general practitioners in Saxony–Anhalt, Germany who had a consultation between May-June 2005	Validated German version of the Patient Health Questionnaire (PHQ) based on DSM-IV diagnostic criteria for depression.	Asthma	Depression at baseline was associated with unscheduled hospital admission within the following year during follow-up (OR 6.1; 95% CI 1.5–24.6, p = 0.011).
**Shah et al 2018** [39]	2010–2014	USA	Retrospective observational	25,259	Adult patients with the primary discharge diagnosis of chronic pancreatitis on the Nationwide Readmission Database (NRD)	ICD-9-CM codes for depression.	Chronic pancreatitis	Depression predicted 30-day readmission to hospital (HR, 1.17; 95% CI, 1.10–1.25)
**Sokoreli et al 2018** [40]	2012–2016	UK	Prospective cohort	671	Patients hospitalised for heart failure enrolled in OPERA-HF observational study cohort	Hospital Anxiety and Depression Scale (HADS)—2 groups with cut-off of 8+ points.	Heart failure	Individuals with moderate-to-severe depression were significantly at greater risk of first unplanned readmission (HR = 1.73, 95%CI 1.24–2.41) and recurrent events (HR-1.76, 95%CI 1.25–2.47), compared to those with none-to-mild depression.
**Xu et al 2008** [41]	2004–2006	China	Prospective cohort	491	Patients aged 30+ with a diagnosis of COPD across 10 general hospitals in Beijing, China	Hospital Anxiety and Depression Scale (HADS), with 2 groups with cut-off score 7+	COPD	A higher HADS depression score > 11 was associated with an increased risk of hospital admission (aIRR 1.72, 95% CI 1.04–2.85), symptom-based exacerbations (aIRR, 1.51; 95% CI 1.01–2.24) and event-based exacerbations (adjusted IRR 1.56; 95% CI, 1.02–2.40) compared with those with lower depression scores < 7.

**Abbreviations**: Adjusted Incidence Rate Ratio = aIRR, Adjusted Odds Ratio = aOR, Center for Epidemiologic Studies Depression Scale = CES-D, Confidence Interval = CI, Emergency Department = ED, Hospital Anxiety and Depression Scale (HADS), Hazard ratio = HR, International Classification of Diseases 9^th^ Version Clinical Modification = ICD-9-CM, International Classification of Diseases 10^th^ Version = ICD-10, Odds Ratio = OR, Patient Health Questionnaire = PHQ, Prevalence Ratio = PR, Risk Ratio = RR.

### Study characteristics

17 studies were based in high-income settings including USA (n = 12) [24, 27, 29–37, 39], UK (n = 4) [17, 21, 25, 40] and Germany (n = 1) [38], while three studies were based in low-to-middle income countries including Mexico [26], Iran [28], and China [41]. All studies employed cross-sectional or observational study designs, including prospective cohort, retrospective and longitudinal observational studies. No randomised controlled studies were found. Sample sizes ranged from 157 to 469,368 patients, with the majority (n = 11) reporting on >1000 participants. Sample populations mainly comprised patients from outpatient clinics in hospitals (n = 7) [24, 26–28, 33, 40, 41], primary care (n = 6) [17, 21, 25, 30, 34, 38], existing databases or registries (n = 6) [29, 31, 32, 35, 37, 39], and one survey [36]. hospitals (n = 7) [24, 26–28, 33, 40, 41], primary care (n = 6) [17, 21, 25, 30, 34, 38], existing databases or registries (n = 6) [29, 31, 32, 35, 37, 39], and one survey [36].

### Risk of bias across studies

All studies clearly defined their objective and study population; recruited participants from the same population with uniform eligibility criteria applied; clearly defined and implemented both exposure and outcome measures consistently across study participants; measured and adjusted for key potential confounders (see S3 Table). All but one study [38] measured the exposure (i.e., depression status) only once in the study period. Most studies (n = 14) measured the exposure of interest before the outcome; the six that did not were cross-sectional studies. Only five studies measured varying levels of exposure, namely various categories of depression severity as per the validated instrument.

### Depression measures

Various methods and tools were used to ascertain depression status in studies’ samples. This included depression scales (n = 6) such as the Hospital Anxiety and Depression Scale (HADS) [21, 25, 28, 40, 41] and Centre for Epidemiologic Studies Depression Scale (CES-D) [27], and patient surveys with a depressive symptom component such as Patient Health Questionnaire-9 (PHQ-9) (n = 4) [24, 30, 34, 38]. Other studies used medical records to confirm depression diagnosis (n = 2) [17, 26]. Cross-sectional or retrospective observational studies using larger patient databases used diagnostic codes for depression from the International Classification of Diseases (ICD) (n = 8) [29, 31–33, 35–37, 39].

### Index comorbidities

Index comorbidities refer to the main condition of focus in each study, given that participants had at least two simultaneous chronic conditions. A range of comorbidities were observed across the included studies (Table 1); the most frequently studied index condition was cancer (n = 4) followed by chronic obstructive pulmonary disease (COPD) (n = 3), heart failure (n = 3), asthma (n = 2), diabetes (n = 2), chronic inflammatory conditions (chronic pancreatitis and ulcerative colitis, n = 2), and HIV (n = 1). Three studies demonstrated a broader scope of more than one comorbidity amongst study participants in their analysis of the association of whether depression predicted emergency hospital admissions [17, 21, 29]. One study investigated 19 conditions as comorbidities alongside depression which were based on the UK Quality Outcomes Framework (QOF) for General Practitioners (GPs) [17].

### Depression and unplanned secondary healthcare

All 20 studies considered emergency hospital admissions as an outcome, while nine also looked at emergency department (ED) visits [24, 26, 27, 29, 30, 33–36] and four studies also looked at emergency readmissions [33, 37, 39, 40]. Across the majority of the studies, depression was positively associated with increased emergency department visits [26, 29, 30, 33–36], emergency hospital admissions [17, 21, 24, 25, 27–41] and emergency hospital readmissions [33, 37, 39, 40] (Table 1).

#### Emergency department (ED) visits

Seven out of nine studies that studied ED visits as an outcome demonstrated that depression predicted increased visits e.g., OR = 2.45 in cancer patients [33] and HR = 1.83 in coronary artery disease patients [29]. However, having depression was not significantly associated with ED visits among patients with heart failure [24] and asthma [27].

#### Emergency hospital admissions

Depression predicted emergency hospital admissions in 19 out of 20 studies; some studies demonstrated that this positive association was proportional to increasing increments of depression severity [21, 24, 25, 34, 40, 41]. Table 2 demonstrates this finding in studies utilising depression instruments like HADS or PHQ-9 to produce score-based patient categories. The higher the depression score the greater the risk of having an emergency hospital admission [21, 24, 25, 34, 40, 41]. Individuals with moderate-to-severe depressive symptoms had a 70% greater risk for all-cause admissions compared to those without symptoms, while those with mild depressive symptoms had a 57% risk when compared to patients without depression [24]. Similarly, having a HADS depression score of 8+, namely moderate-to-severe depression, was associated with 4.8 times higher risk of emergency hospital admission, while having a HADS score between 4–7 corresponding to subthreshold depression was associated with 2.8 times higher odds compared to individuals without depression [25]. Across the studies, depression predicted the greatest magnitude of risk, namely 6.1 times greater, among patients with asthma [38].

**Table 2:** Gradient of association between scale-based depression sub-groups and unplanned secondary healthcare outcomes.

Study	Measure	Depression Scale	Scale Score	Outcomes				
				*ED visits*	*Emergency hospital admissions*	*Specific-cause emergency admissions*		*Emergency hospital readmissions*
Bhatt et al. 2016 [24]	RR	PHQ-9	Minimal (0–4) (Ref)	1	1	HF-related	1	-
			Mild (5–9)	1.14	1.57*		2.17	-
			Moderate-severe (10–27)	1.5	1.70*		2.50*	-
Blakemore et al. 2019 [25]	OR	HADS	0–3 (Ref)	1	1	-		-
			4–7	2.40*	2.84*	-		-
			8–11	4.56**	4.80**	-		-
			12+	4.65**	4.82**	-		-
Guthrie et al. 2016 [21]	OR	HADS	0–1 (Ref)	-	1	-		-
			2–4	-	0.99	-		-
			5–7	-	1.73	-		-
			8–10	-	1.67	-		-
			11+	-	2.42*	-		-
Moraska et al. 2013 [34]	HR	PHQ-9	None-minimal (0–4) (Ref)	1	1	-		-
			Mild (5–9)	1.35**	1.16	-		-
			Moderate-severe (10+)	1.83**	1.79**	-		-
Sokorelli et al. 2018 [40]	HR	HADS	None-to-mild (Ref)	-	-	-		1
			Moderate-to-severe	-	-	-		1.74*
Xu et al. 2008 [41]	IRR	HADS	No depression (0–7) (Ref)	-	1	COPD-event	1	-
			Possible depression (8–10)	-	1.37		1.30	-
			Probable depression (11+)	-	1.72*		1.56*	-

(Green) Statistically significant positive effect [*p<0.05, **p<0.001]; (darker shades correspond to greater magnitude of risk)

(White) No statistically significant effect

(-) Outcome not studied

**Abbreviations**: COPD, Chronic Obstructive Pulmonary Disease; HADS, Hospital Anxiety and Depression Scale [Depression score]; HF, Heart Failure; HR, Hazard Ratio; IRR, Incidence Rate Ratio; OR, Odds Ratio; PHQ-9, Patient Health Questionnaire-9; RR, Risk Ratio.

**Note**: The measures of association show adjusted findings, controlling for various sociodemographic factors in the respective studies.

The reasons for emergency hospital admissions were not necessarily mentioned across all studies, although some distinguished admissions as being specific to the index comorbidities studied. For example, Bhatt and colleagues [24] report heart failure-related admissions as well as all-cause admissions; both outcomes showed statistically significant greater risks in patients with depression, namely 70% for all-cause compared to 57% for heart failure-related admissions. Other studies specified COPD exacerbations [25, 28, 41] or severe hypoglycaemic episodes in diabetes patients requiring emergency hospital admissions [26, 30] as potential reasons for increased use in patients with depression.

In a few of the studies investigating both emergency hospital admissions and ED visits, depression predicted one outcome but not the other [25–27]. For example, having depression was significantly associated with a higher rate of ED use in patients with type 1 diabetes but it did not significantly predict emergency hospital admissions that were diabetes-related such as severe hypoglycaemic episodes [26]. However, in a larger diabetes population, depression not only significantly predicted a 42% higher risk of first hypoglycaemic episode requiring emergency hospital admission but also 34% greater risk of these events occurring over 5 years [30].

#### Emergency hospital readmissions

Four studies investigated the risk of readmission in patients with various multimorbidity profiles and depression, which all reported a statistically significant greater risk for readmission in those with depression [33, 37, 39, 40]. In patients with heart failure, moderate-to-severe depression predicted a 73% higher risk of first unplanned readmission [40] than for patients without depression or only mild depression. Among patients with chronic pancreatitis, those with co-morbid depression had a 17% greater risk of 30-day readmission [39], while in ulcerative colitis patients this risk was 40% higher in patients with depression [37]. On the other hand, 30-day readmissions were 2.03 times more likely in cancer patients with depression compared to those without depression [33].

### Sociodemographic predictors of unplanned secondary healthcare use

Although most studies adjusted results for sociodemographic covariates, only one study [31] reported stratified risk estimates for a sociodemographic predictor of unplanned secondary healthcare use in patients with both multimorbidity and depression. Females had a statistically significant higher risk of an emergency hospital admission than males among individuals aged 50+ with depression and diagnosed head-neck cancer [31]. Though significant, this difference between the sexes was small, with 34% higher risk for women versus 28% higher risk for men when comparing patients with and without depression in sub-analyses [31]. The effect of sex was not reported as a stratified result nor was the interaction with depression explored in multimorbid patients in the rest of the included papers. Another study showed older age in multimorbid patients with depression positively correlated with an increasing proportion of all patients accessing unplanned care over a year [17].

### Risk stratification by multimorbidity clusters

No studies analysed the risk of any type of unplanned secondary healthcare use by multimorbidity disease combinations or clusters; index conditions were individually studied without further exploration of the cumulative effects of multiple conditions in the same individuals. Though not a risk estimate, one study showed that the number of long-term conditions were positively correlated with a greater percentage of patients using unplanned secondary care [17]. Those with 2+ conditions comprised 25%, while 3+ conditions made up 35% of the total population [17].

## Discussion

### Summary of findings

This review found that presence of depression increases the likelihood of emergency hospital admissions and readmissions in patients with multimorbidity. This association holds across a range of long-term conditions characterising multimorbidity in various countries, settings and samples. Depression also predicted increased ED visits in most of the studies reporting on this outcome. Moreover, the greater the severity of depression, the greater the risk of emergency hospital admissions and ED visits.

Patients with co-occurring depression with cancers [29], COPD [25], and asthma [38] showed some of the greatest magnitudes of risk of unplanned secondary healthcare use. Being female [31], of older age [17] and having a greater number of long-term conditions [17] were other predictors of unplanned secondary healthcare use.

### Strengths and limitations

This review captures a broad scope of multimorbidity, inclusive of any chronic comorbidity, in an area of growing importance and contributes up-to-date evidence that adds to a previous systematic review. We focussed on unplanned secondary healthcare use instead of all types of unplanned care, such as urgent GP home visits, use of walk-in centres and minor injuries units, which allowed for more uniform comparison and synthesis of study findings. We conducted an extensive search of six electronic databases with a comprehensive search strategy designed to capture a broad range of multimorbidity studies without limiting comorbidity, publication language, sample size or follow-up duration. Our review additionally highlights the effect of incremental depression severity on the studied association, beyond establishing a simple association. Through excluding studies that recruited individuals from the emergency department itself or during an active inpatient period, we aimed to minimise confounding as much as possible.

However, some limitations exist. Firstly, the range of different conditions included in the review may have inherently different effects to each other on the studied outcome, based on whether some conditions are more prone to adverse events requiring greater emergency care episodes than others. For example, conditions like COPD or heart failure have varying severity levels which are highly sensitive to exacerbations or adverse events, compared to other conditions like hypertension or diabetes where effects may be relatively slower to accumulate. Secondly, the use of depression scales in some studies only measures status and severity of depression cross-sectionally, which does not capture the onset and type of depression, namely episodic versus recurrent. We are not able to deduce what the temporal effect of depression is based on duration of illness before and during study periods to consider the bidirectional relationship with long-term conditions. Thirdly, sample sizes and follow-up periods varied across the studies which may explain some of the variation in the effect sizes. For example, two studies reporting diabetes-related emergency admissions conclude opposite findings which may be related to sample size and outcome duration of hypoglycaemic episodes due to different follow-up periods [26, 30]. Also, we did not conduct a meta-analysis due to large dissimilarity of study designs and outcome measures.

### Comparison with the literature

This review concurs with the overall positive association between depression and unplanned secondary healthcare found in the previous review on all types of unscheduled care for four selected comorbidities [22]. The previous review indicated a 49% greater odds of unscheduled care associated with having depression [22]; however, this effect was smaller and non-significant when covariates were controlled for. Our review did not restrict the number or type of comorbidity in the reviewed studies, in contrast to only four long-term conditions included in the previous review [22]. However, both reviews demonstrate the positive association is valid across a range of conditions across different studies, including those published since 2012 [17, 21, 24–26, 30–37, 39, 40]. The limitation in both reviews is that there is limited evidence that compares combinations of more than two conditions, to reflect the broader multimorbidity spectrum in the general population with comorbid depression.

We found that incremental increases in depression severity scores were associated with an increase in the magnitude of risk of unplanned secondary care outcomes, particularly for COPD [25] and heart failure [24, 34]. However, the effects of depression were modest yet statistically significant across most studies included. In primary care, depending on patient age and disease combination, depression has multiplicative effects translating to higher patient costs [12, 14]. Whether the effects of depression with comorbidities in this review were additive or multiplicative are inconclusive, in the context of unplanned secondary care. This requires further investigation of more complex disease combinations with depression. Recent evidence shows that multimorbidity combinations with the highest costs per patient (which have depression in the top two combinations) do not necessarily incur the highest total costs for unplanned secondary care [42], thus making it challenging to identify conclusive patterns on the impact of depression with other conditions and different numbers of conditions for patient outcomes.

### Significance of findings and implications

The strong association between increased depression severity and increased unplanned secondary healthcare use suggests limitations in the adequacy of planned care and ongoing management of physical comorbidity in individuals with co-occurring depression. Particular attention must be paid to those with high depression scores to ensure the depression is optimally treated, to review clinical management plans, and utilise other resources such as integrated multidisciplinary teams to reduce avoidable unplanned care [43]. Depression screening should be performed amongst patients with multimorbidity due to an increased risk of depression, to identify those may be at risk of unplanned secondary healthcare. Those who exhibit greater severity of depression should be reviewed for risk of emergency hospital admissions using validated tools, which can facilitate early detection of at-risk patients with multimorbidity [44]. Similarly, patients with mild depression may benefit from regular reviews as a preventative strategy to plan care needs and treatment effectively before exacerbation of depression symptoms [45]. Such approaches require careful consideration of whether a patient has episodic or recurrent depression, which may impact unplanned care differently. However, the treatment of depression does not guarantee immediate improvements in unplanned care use across the board; understanding the effects of depression on the severity of co-occurring conditions over longer durations and interaction of common risk factors is crucial. Thus, care plans must consider the continuity of care relating to the management of moderate-to-severe depression and integrate primary care interventions that target risk factors driving worsening depression for patients with multimorbidity. Moreover, evaluations of different community-based interventions to specifically manage patients with multimorbidity and depression are needed to understand which interventions may be effective in preventing excess unplanned secondary care use.

### Future research

This review suggests a gap in our understanding of how the risk of using unplanned secondary healthcare may vary with multimorbidity clusters i.e., the broader spectrum of disease combinations that make up patients’ multimorbidity. This is important to investigate in patients, particularly those with complex multimorbidity which is becoming more common, namely those with more than two conditions belonging to multiple body systems. Additionally, there is limited experimental evidence on whether interventions to treat depression in people with multimorbidity reduce unplanned care use. Nor is there sufficient evidence on whether depression interacts with certain patient characteristics to exacerbate the risk of emergency hospital admissions among individuals with multimorbidity. This gap requires investigating the stratified risk for different patient groups and their heterogenous multimorbidity profiles in future research. Furthermore, future research should seek to understand the reasons for increased emergency care in patients with depression, using mixed methods approaches.

## Conclusions

Concurrent depression is an important predictor of unplanned secondary healthcare use in individuals with multimorbidity, with this effect appearing to increase with greater severity of depression. More research is needed to identify multimorbidity clusters and other patient characteristics which may predict unplanned care use as well as the reasons for this effect in depression. Individuals with depression and multimorbidity should be proactively identified in the primary care setting and action taken (through depression severity and admission risk scoring tools) to identify those at particularly high risk of unplanned care use, to conduct regular primary care reviews, assessment, and treatment to reduce this risk.

## Supporting information

S1 Table Preferred Reporting Items for Systematic Reviews and Meta-Analysis (PRISMA) 2020 checklist. (DOCX)

S2 Table Literature search strategy for all electronic databases. Search terms and combinations for Medline, Embase, PsychINFO, Web of Science, CENTRAL, CINAHL databases, performed in March 2021.
(DOCX)

S3 Table Quality assessment (risk of bias) of the included studies in the systematic review. Results using the National Heart, Lung, and Blood Institute (NHBLI) Study Quality Assessment Tools (Available at: https://www.nhlbi.nih.gov/health-topics/study-quality-assessment-tools). **Legend**: Yes (Y); No (N); Other (Not Applicable (N/A); Not Reported (N/R); Cannot Determine (C/D)). **Overall Rating**: Good, Fair, Poor.
(DOCX)
