# Supplementary material for: Depression and unplanned secondary healthcare use in patients with multimorbidity: A systematic review
Source: PLoS One. 2022 Apr 7;17(4):e0266605. doi: 10.1371/journal.pone.0266605 (PMC8989325; doi:10.1371/journal.pone.0266605)
Supplement: S2 Table — Search terms and combinations for Medline, Embase, PsychINFO, Web of Science, CENTRAL, CINAHL databases, performed in March 2021. (DOCX) [file pone.0266605.s002.docx]

**S2 Table. Literature search strategy for all electronic databases -** Search terms and combinations for Medline, Embase, PsychINFO, Web of Science, CENTRAL, CINAHL databases, performed in March 2021.

| **MEDLINE**   1. depression/ 2. (depress* or dysthymi* or mood? or affective disorder* or affective symptom*).mp. 3. or/1-2 4. exp comorbidity/ or exp multimorbidity/ 5. (comorbid* or co-morbid*).mp. 6. (multimorbid* or multi-morbid*).mp. 7. (multidisease? or multi-disease? or (multiple adj (ill* or disease? or condition? or syndrom* or disorder?))).mp. 8. ((cooccur* or co-occur* or coexist* or co-exist* or multipl*) adj3 (disease? or ill* or care or condition? or disorder* or health* or symptom* or syndrom*)).mp. 9. exp chronic disease/ 10. (chronic* adj3 (disease? or ill* or care or condition? or disorder* or health* or syndrom* or symptom*)).mp. 11. or/4-10 12. exp diabetes mellitus/ or diabet*.mp. 13. exp hypertension/ or (hypertens* or "high blood pressure?").mp. 14. exp heart diseases/ or (((heart or cardiac or cardiovascular or coronary) adj (disease? or disorder? or failure)) or arrythmia?).mp. 15. exp cerebrovascular disorders/ or ((cerebrovascular or vascular or carotoid* or arter*) adj (disorder? or disease?)).mp. 16. exp asthma/ or asthma*.mp. 17. exp pulmonary disease chronic obstructive/ or (copd or (pulmonary adj2 (disease? or disorder?))).mp. 18. exp hyperlipidemia/ or (hyperlipidem* or hypercholesterolemi* or hypertriglyceridemi*).mp. 19. exp thyroid diseases/ or ((thyroid adj (disease? or disorder)) or hyperthyroid* or hypothyroid*).mp. 20. exp arthritis rheumatoid/ or rheumatoid arthritis.mp. 21. exp epilepsy/ or (epileps* or seizure?).mp. 22. exp hiv infections/ or (HIV or acquired immune* deficiency syndrome? or (aids adj (associated or related or arteritis))).mp. 23. exp neoplasms/ or (neoplasm? or cancer?).mp. 24. exp kidney diseases/ or (kidney adj (disease? or disorder?)).mp. 25. exp liver diseases/ or (liver adj (disease? or disorder?)).mp. 26. exp osteoporosis/ or osteoporosis.mp. 27. or/12-26 28. 3 and 11 29. 3 and 27 30. 28 or 29 31. exp Emergency Medical Services/ 32. exp Emergency Service, Hospital/ 33. ((unscheduled or unplanned or emergency or urgent or "accident and emergency" or A&E or casualty department* or emergency department* or emergency ward* or emergency room* or ER) adj2 (care or visit* or attendance* or admission* or hospitali?ation*)).mp. 34. ((urgent or unplanned or unscheduled or emergency) adj2 (readmission* or re-admission* or rehospitali?ation* or re-hospitali?ation*)).mp. 35. or/31-34 36. 30 and 35 37. limit 36 to yr="2000 - 2021"   .mp. = title, original title, abstract, name of substance word, subject heading word  / = MeSH term  exp = explode all terms under searched MeSH term (if explode available)  adjN = term proximity operator |
| --- |
| **EMBASE**   1. exp depression/ or exp chronic depression/ or exp major depression/ 2. (depress* or dysthymi* or mood? or affective disorder* or affective symptom*).mp. 3. or/1-2 4. exp comorbidity/ or exp multiple chronic conditions/ 5. (comorbid* or co-morbid*).mp. 6. (multimorbid* or multi-morbid*).mp. 7. (multidisease? or multi-disease? or (multiple adj (ill* or disease? or condition? or syndrom* or disorder?))).mp. 8. ((cooccur* or co-occur* or coexist* or co-exist* or multipl*) adj3 (disease? or ill* or care or condition? or disorder* or health* or symptom* or syndrom*)).mp. 9. exp chronic disease/ 10. (chronic* adj3 (disease? or ill* or care or condition? or disorder* or health* or syndrom* or symptom*)).mp. 11. or/4-10 12. exp diabetes mellitus/ or diabet*.mp. 13. exp hypertension/ or (hypertens* or "high blood pressure?").mp. 14. exp heart disease/ or exp myocardial disease/ or (((heart or cardiac or cardiovascular or coronary) adj (disease? or disorder? or failure)) or arrythmia?).mp. 15. exp cerebrovascular disease/ or exp carotid artery disease/ or ((cerebrovascular or vascular or carotoid* or arter*) adj (disorder? or disease?)).mp. 16. exp asthma/ or asthma*.mp. 17. exp Chronic Obstructive Lung Disease/ or (copd or (pulmonary adj2 (disease? or disorder?))).mp. 18. exp hyperlipidemia/ or exp hypercholesterolemia/ or (hyperlipidem* or hypercholesterolemi* or hypertriglyceridemi*).mp. 19. exp thyroid disease/ or ((thyroid adj (disease? or disorder)) or hyperthyroid* or hypothyroid*).mp. 20. exp rheumatoid arthritis/ or rheumatoid arthritis.mp. 21. exp epilepsy/ or (epileps* or seizure?).mp. 22. exp Human Immunodeficiency Virus/ or (HIV or acquired immune* deficiency syndrome? or (aids adj (associated or related or arteritis))).mp. 23. exp neoplasm/ or (neoplasm? or cancer?).mp. 24. exp kidney disease/ or (kidney adj (disease? or disorder?)).mp. 25. exp liver disease/ or (liver adj (disease? or disorder?)).mp. 26. exp osteoporosis/ or osteoporosis.mp. 27. or/12-26 28. 3 and 11 29. 3 and 27 30. 28 or 29 31. exp emergency care/ or exp emergency ward/ or exp emergency health service/ or exp hospital emergency service/ 32. ((unscheduled or unplanned or emergency or urgent or "accident and emergency" or A&E or casualty department* or emergency department* or emergency ward* or emergency room* or ER) adj2 (care or visit* or attendance* or admission* or hospitali?ation*)).mp. 33. ((urgent or unplanned or unscheduled or emergency) adj2 (readmission* or re-admission* or rehospitali?ation* or re-hospitali?ation*)).mp. 34. or/31-33 35. 30 and 34 36. limit 35 to yr="2000 - 2021"   .mp. = title, original title, abstract, name of substance word, subject heading word  / = MeSH term  exp = explode all terms under searched MeSH term (if explode available)  adjN = term proximity operator |
| **PsycINFO**   1. exp major depression/ 2. (depress* or dysthymi* or mood? or affective disorder* or affective symptom*).mp. 3. or/1-2 4. exp comorbidity/ 5. (comorbid* or co-morbid*).mp. 6. (multimorbid* or multi-morbid*).mp. 7. (multidisease? or multi-disease? or (multiple adj (ill* or disease? or condition? or syndrom* or disorder?))).mp. 8. ((cooccur* or co-occur* or coexist* or co-exist* or multipl*) adj3 (disease? or ill* or care or condition? or disorder* or health* or symptom* or syndrom*)).mp. 9. exp chronic illness/ 10. (chronic* adj3 (disease? or ill* or care or condition? or disorder* or health* or syndrom* or symptom*)).mp. 11. or/4-10 12. exp diabetes/ or diabet*.mp. 13. exp hypertension/ or (hypertens* or "high blood pressure?").mp. 14. exp heart disorders/ or exp cardiovascular disorders/ or (((heart or cardiac or cardiovascular or coronary) adj (disease? or disorder? or failure)) or arrythmia?).mp. 15. exp cerebrovascular disorders/ or ((cerebrovascular or vascular or carotoid* or arter*) adj (disorder? or disease?)).mp. 16. exp asthma/ or asthma*.mp. 17. exp chronic obstructive pulmonary disease/ or (copd or (pulmonary adj2 (disease? or disorder?))).mp. 18. exp lipid metabolism disorders/ or (hyperlipidem* or hypercholesterolemi* or hypertriglyceridemi*).mp. 19. exp thyroid disorders/ or ((thyroid adj (disease? or disorder)) or hyperthyroid* or hypothyroid*).mp. 20. exp rheumatoid arthritis/ or rheumatoid arthritis.mp. 21. exp epilepsy/ or (epileps* or seizure?).mp. 22. exp HIV/ or (HIV or acquired immune* deficiency syndrome? or (aids adj (associated or related or arteritis))).mp. 23. exp neoplasms/ or (neoplasm? or cancer?).mp. 24. exp kidney diseases/ or (kidney adj (disease? or disorder?)).mp. 25. exp liver disorders/ or (liver adj (disease? or disorder?)).mp. 26. exp osteoporosis/ or osteoporosis.mp. 27. or/12-26 28. 3 and 11 29. 3 and 27 30. 28 or 29 31. exp Emergency Services/ 32. ((unscheduled or unplanned or emergency or urgent or "accident and emergency" or A&E or casualty department* or emergency department* or emergency ward* or emergency room* or ER) adj2 (care or visit* or attendance* or admission* or hospitali?ation*)).mp. 33. ((urgent or unplanned or unscheduled or emergency) adj2 (readmission* or re-admission* or rehospitali?ation* or re-hospitali?ation*)).mp. 34. or/31-33 35. 30 and 34 36. limit 35 to yr="2000 - 2021"   .mp. = title, original title, abstract, name of substance word, subject heading word  / = MeSH term  exp = explode all terms under searched MeSH term (if explode available)  adjN = term proximity operator |
| **Web of Science**   1. TS=(depress* or dysthymi* or mood? or affective disorder* or affective symptom*) 2. TS=(comorbid* or co-morbid* or multimorbid* or multi-morbid* or multidisease? or multi-disease?) 3. TS=(multiple NEAR/1 (ill* or disease? or condition? or syndrom* or disorder?)) 4. TS=((cooccur* or co-occur* or coexist* or co-exist* or multipl*) NEAR/3 (disease? or ill* or care or condition? or disorder* or health* or symptom* or syndrom*)) 5. TS=(chronic* NEAR/3 (disease? or ill* or care or condition? or disorder* or health* or syndrom* or symptom*)) 6. TS=(diabet*) 7. TS=(hypertens* or "high blood pressure?") 8. TS=(((heart or cardiac or cardiovascular or coronary) NEAR/1 (disease? or disorder? or failure)) or arrythmia?) 9. TS=((cerebrovascular or vascular or carotoid* or arter*) NEAR/1 (disorder? Or disease?)) 10. TS=(asthma*) 11. TS=(COPD or (pulmonary NEAR/2 (disease? or disorder?))) 12. TS=(hyperlipidem* or hypercholesterolemi* or hypertriglyceridemi*) 13. TS=((thyroid NEAR/1 (disease? or disorder)) or hyperthyroid* or hypothyroid*) 14. TS=(rheumatoid arthritis or arthritis) 15. TS=(epileps* or seizure?) 16. TS=(HIV or acquired immune* deficiency syndrome? or (aids NEAR/1 (associated or related or arteritis))) 17. TS=(neoplasm? or cancer?) 18. TS=(kidney NEAR/1 (disease? or disorder?)) 19. TS=(liver NEAR/1 (disease? or disorder?)) 20. TS=(osteoporosis) 21. TS=((unscheduled or unplanned or emergency or urgent or "accident and emergency" or A&E or "casualty department*" or "emergency department*" or "emergency ward*" or "emergency room*" or ER) NEAR/2 (care or visit* or attendance* or admission* or hospitali?ation*)) 22. TS=((urgent or unplanned or unscheduled or emergency) NEAR/2 (readmission* or re-admission* or rehospitali?ation* or re-hospitali?ation*)) 23. #5 OR #4 OR #3 OR #2 24. #20 OR #19 OR #18 OR #17 OR #16 OR #15 OR #14 OR #13 OR #12 OR #11 OR #10 OR #9 OR #8 OR #7 OR #6 25. #23 AND #1 26. #24 AND #1 27. #26 OR #25 28. #22 OR #21 29. #28 AND #27 30. #29 AND PY=(2000-2021)   TS= Topic search. Searches the Topic fields in all databases, including Titles, Abstracts, Keywords and Indexing fields such as Systematics, Taxonomic Terms and Descriptors. |
| **Cochrane Library Central Register of Controlled Trials (CENTRAL)**   1. (depression) 2. MeSH descriptor: [Depression] explode all trees 3. #1 OR #2 4. MeSH descriptor: [Comorbidity] explode all trees 5. MeSH descriptor: [Multimorbidity] explode all trees 6. (comorbid*) or (co-morbid*) 7. (multimorbid*) or (multi-morbid*) 8. (multidisease?) or (multi-disease?) 9. (multiple NEAR/1 (ill* or disease? or condition? or syndrom* or disorder?)) 10. ((cooccur* or co-occur* or coexist* or co-exist* or multipl*) NEAR/3 (disease? or ill* or care or condition? or disorder* or health* or symptom* or syndrom*)) 11. MeSH descriptor: [Chronic Disease] explode all trees 12. (chronic* NEAR/3 (disease? or ill* or care or condition? or disorder* or health* or syndrom* or symptom*)) 13. #4 OR #5 OR #6 OR #7 OR #8 OR #9 OR #10 #11 OR #12 14. (diabet*) 15. MeSH descriptor: [Diabetes Mellitus] explode all trees 16. (hypertens*) or ("high blood pressure?") 17. MeSH descriptor: [Hypertension] explode all trees 18. (((heart or cardiac or cardiovascular or coronary) NEAR/1 (disease? or disorder? or failure)) or arrythmia?) 19. MeSH descriptor: [Heart Diseases] explode all trees 20. ((cerebrovascular or vascular or carotoid* or arter*) NEAR/1 (disorder? or disease?)) 21. MeSH descriptor: [Cerebrovascular Disorders] explode all trees 22. (asthma*) 23. MeSH descriptor: [Asthma] explode all trees 24. (copd or (pulmonary NEAR/2 (disease? or disorder?))) 25. MeSH descriptor: [Pulmonary Disease, Chronic Obstructive] explode all trees 26. (hyperlipidem* or hypercholesterolemi* or hypertriglyceridemi*) 27. MeSH descriptor: [Hyperlipidemias] explode all trees 28. ((thyroid adj (disease? or disorder)) or hyperthyroid* or hypothyroid*) 29. MeSH descriptor: [Thyroid Diseases] explode all trees 30. (rheumatoid arthritis) 31. MeSH descriptor: [Arthritis, Rheumatoid] explode all trees 32. (epileps* or seizure?) 33. MeSH descriptor: [Epilepsy] explode all trees 34. (HIV or acquired immune* deficiency syndrome? or (aids NEAR/1 (associated or related or arteritis))) 35. MeSH descriptor: [HIV] explode all trees 36. (neoplasm? or cancer?) 37. MeSH descriptor: [Neoplasms] explode all trees 38. (kidney NEAR/1 (disease? or disorder?)) 39. MeSH descriptor: [Kidney Diseases] explode all trees 40. (liver NEAR/1 (disease? or disorder?)) 41. MeSH descriptor: [Liver Diseases] explode all trees 42. (osteoporosis) 43. MeSH descriptor: [Osteoporosis] explode all trees 44. #14 OR #15 OR #16 OR #17 OR #18 OR #19 OR #20 OR #21 OR #22 OR #23 OR #24 OR #25 OR #26 OR #27 OR #28 OR #29 OR #30 OR #31 OR #32 OR #33 OR #34 OR #35 OR #36 OR #37 OR #38 OR #39 OR #40 OR #41 OR # 42 OR #43 45. #3 AND #13 46. #3 AND #44 47. #45 OR #46 48. MeSH descriptor: [Emergency Service, Hospital] explode all trees 49. MeSH descriptor: [Emergency Medical Services] explode all trees 50. ((unscheduled or unplanned or emergency or urgent or "accident and emergency" or A&E or casualty department* or emergency department* or emergency ward* or emergency room* or ER) NEAR/2 (care or visit* or attendance* or admission* or hospitali?ation*)) 51. ((urgent or unplanned or unscheduled or emergency) NEAR/2 (readmission* or re-admission* or rehospitali?ation* or re-hospitali?ation*)) 52. #48 OR #49 OR #50 OR #51 53. #47 AND #52 54. #47 AND #52 with Publication Year from 2000 to 2021, in Trials |
| **CINAHL**   1. TX (depress* or dysthymi* or mood? or affective disorder* or affective symptom*) 2. (MH "Depression+") 3. S1 OR S2 4. (MH "Comorbidity") 5. TX (comorbid* or co-morbid*) 6. TX (multimorbid* or multi-morbid*) 7. TX (multidisease? or multi-disease? or (multiple N1 (ill* or disease? or condition? or syndrom* or disorder?))) 8. TX ((cooccur* or co-occur* or coexist* or co-exist* or multipl*) N3 (disease? or ill* or care or condition? or disorder* or health* or symptom* or syndrom*)) 9. (MH "Chronic Disease+") 10. (chronic* N3 (disease? or ill* or care or condition? or disorder* or health* or syndrom* or symptom*)) 11. S4 OR S5 OR S6 OR S7 OR S8 OR S9 OR S10 12. TX (diabet*) 13. (MH "Diabetes Mellitus+") 14. TX (hypertens* or "high blood pressure?") 15. (MH "Hypertension+") 16. TX (((heart or cardiac or cardiovascular or coronary) N1 (disease? or disorder? or failure)) or arrythmia?) 17. (MH "Cardiovascular Diseases+") 18. TX ((cerebrovascular or vascular or carotoid* or arter*) N1 (disorder? or disease?)) 19. (MH "Cerebrovascular Disorders+") 20. TX (asthma*) 21. (MH "Asthma+") 22. TX (copd or (pulmonary N2 (disease? or disorder?))) 23. (MH "Pulmonary Disease, Chronic Obstructive+") 24. TX (hyperlipidem* or hypercholesterolemi* or hypertriglyceridemi*) 25. (MH "Hyperlipidemia+") 26. TX ((thyroid N1 (disease? or disorder)) or hyperthyroid* or hypothyroid*) 27. (MH "Thyroid Diseases+") 28. TX (rheumatoid arthritis) 29. (MH "Arthritis, Rheumatoid+") 30. TX (epileps* or seizure?) 31. (MH "Epilepsy+") 32. TX (HIV or acquired immune* deficiency syndrome? or (aids N1 (associated or related or arteritis))) 33. (MH "Human Immunodeficiency Virus+") 34. TX (neoplasm? or cancer?) 35. (MH "Neoplasms+") 36. TX (kidney N1 (disease? or disorder?)) 37. (MH "Kidney Diseases+") 38. TX (liver N1 (disease? or disorder?)) 39. (MH "Liver Diseases+") 40. TX (osteoporosis) 41. (MH "Osteoporosis+") 42. S12 OR S13 OR S14 OR S15 OR S16 OR S17 OR S18 OR S19 OR S20 OR S21 OR S22 OR S23 OR S24 OR S25 OR S26 OR S27 OR S28 OR S29 OR S30 OR S31 OR S32 OR S33 OR S34 OR S35 OR S36 OR S37 OR S38 OR S39 OR S40 OR S41 43. S3 AND S11 44. S3 AND S42 45. S43 OR S44 46. (MH "Emergency Service+") 47. (MH "Emergency Medical Services+") 48. TX ((unscheduled or unplanned or emergency or urgent or "accident and emergency" or A&E or casualty department* or emergency department* or emergency ward* or emergency room* or ER) N2 (care or visit* or attendance* or admission* or hospitali?ation*)) 49. TX ((urgent or unplanned or unscheduled or emergency) N2 (readmission* or re-admission* or rehospitali?ation* or re-hospitali?ation*)) 50. S46 OR S47 OR S48 OR S49 51. S45 AND S50 52. S51 AND EM 20000101-(retrieve articles entered from January 2000 to the present)   S = Search (on CINAHL)  TX = All Text  MH = CINAHL Exact Subject Heading  + = Explode Subject Heading  EM = Article entry date |
