# Supplementary material for: Depression and unplanned secondary healthcare use in patients with multimorbidity: A systematic review
Source: PLoS One. 2022 Apr 7;17(4):e0266605. doi: 10.1371/journal.pone.0266605 (PMC8989325; doi:10.1371/journal.pone.0266605)
Supplement: S3 Table — Results using the National Heart, Lung, and Blood Institute (NHBLI) Study Quality Assessment Tools (Available at: https://www.nhlbi.nih.gov/health-topics/study-quality-assessment-tools). Legend: Yes (Y); No (N); Other (Not Applicable (N/A); Not Reported (N/R); Cannot Determine (C/D)). Overall Rating: Good, Fair, Poor. (DOCX) [file pone.0266605.s003.docx]

**S3 Table. Quality Assessment (Risk of Bias) of the included studies in the systematic review**. Results using the National Heart, Lung, and Blood Institute (NHBLI) Study Quality Assessment Tools (Available at: <https://www.nhlbi.nih.gov/health-topics/study-quality-assessment-tools>).

| **Author** | **Study Design** | **Q1** | **Q2** | **Q3** | **Q4** | **Q5** | **Q6** | **Q7** | **Q8** | **Q9** | **Q10** | **Q11** | **Q12** | **Q13** | **Q14** | **Overall Rating** |
| --- | --- | --- | --- | --- | --- | --- | --- | --- | --- | --- | --- | --- | --- | --- | --- | --- |
| Bhatt et al 2016 [1] | Prospective cohort | Y | Y | Y | Y | N | Y | Y | Y | Y | N | Y | Y | Y | Y | Good |
| Blakemore et al 2019 [2] | Longitudinal cohort | Y | Y | Y | Y | Y | Y | Y | Y | Y | N | Y | Y | N/R | Y | Good |
| Doubova et al 2018 [3] | Cross-sectional | Y | Y | Y | Y | Y | N | N | N | Y | N | Y | N/R | N/A | Y | Fair |
| Eisner et al 2005 [4] | Prospective cohort | Y | Y | Y | Y | N | Y | Y | N | Y | N | Y | N/R | N/A | Y | Fair |
| Ghanei et al 2007 [5] | Prospective cohort | Y | Y | N/R | Y | N | Y | Y | N | Y | N | Y | N/R | Y | Y | Fair |
| Guthrie et al 2016 [6] | Prospective cohort | Y | Y | N | Y | Y | Y | Y | Y | Y | N | Y | N | N/R | Y | Good |
| Himelhoch et al 2004 [7] | Cross-sectional | Y | Y | Y | Y | N | N | N | N | Y | N | Y | Y | N/A | Y | Fair |
| Katon et al 2013 [8] | Prospective cohort | Y | Y | Y | Y | N | Y | Y | N | Y | N | Y | Y | Y | Y | Good |
| Laurence et al 2017 [9] | Cross-sectional | Y | Y | Y | Y | Y | N | N | N | Y | N | Y | N/A | N/A | Y | Fair |
| Laurence et al 2019 [10] | Cross-sectional | Y | Y | Y | Y | Y | N | N | N | Y | N | Y | N/A | N/A | Y | Fair |
| Mausbach et al 2017 [11] | Retrospective observational | Y | Y | Y | Y | Y | N | N | N | Y | N | Y | N/A | N/A | Y | Fair |
| Moraska et al 2013 [12] | Prospective cohort | Y | Y | N | Y | N | Y | Y | Y | Y | N | Y | N/R | Y | Y | Good |
| Niazi et al 2018 [13] | Retrospective observational | Y | Y | N/R | Y | Y | Y | Y | N | Y | N | Y | Y | N/R | Y | Good |
| Pan et al 2015 [14] | Cross-sectional | Y | Y | Y | Y | Y | N | Y | N | Y | N | Y | N/A | N/A | Y | Good |
| Poojary et al 2017 [15] | Cross-sectional | Y | Y | Y | Y | N | Y | Y | N | Y | N | Y | N/A | N/A | Y | Good |
| Ricketts et al 2018 [16] | Retrospective observational | Y | Y | Y | Y | Y | Y | Y | N | Y | N | Y | Y | N/A | Y | Good |
| Schneider et al 2008 [17] | Longitudinal observational | Y | Y | Y | Y | N | Y | Y | N | Y | Y | Y | Y | N | Y | Good |
| Shah et al 2018 [18] | Retrospective observational | Y | Y | Y | Y | Y | Y | Y | N | Y | N | Y | Y | N/R | Y | Good |
| Sokoreli et al 2018 [19] | Prospective cohort | Y | Y | Y | Y | N | Y | Y | Y | Y | N | Y | C/D | N | Y | Good |
| Xu et al 2008 [20] | Prospective cohort | Y | Y | N/R | Y | Y | Y | Y | N | Y | N | Y | Y | Y | Y | Good |

**Legend:** Yes (Y); No (N); Other (Not Applicable (N/A); Not Reported (N/R); Cannot Determine (C/D)). **Overall Rating:** Good, Fair, Poor

**References:**

1. Bhatt KN, Kalogeropoulos AP, Dunbar SB, Butler J, Georgiopoulou VV. Depression in heart failure: Can PHQ-9 help? Int J Cardiol. 2016;221: 246–250. doi:10.1016/j.ijcard.2016.07.057
2. Blakemore A, Dickens C, Chew-Graham CA, Afzal CW, Tomenson B, Coventry PA, et al. Depression predicts emergency care use in people with chronic obstructive pulmonary disease: a large cohort study in primary care. Int J Chron Obstruct Pulmon Dis. 2019;14: 1343–1353. doi:10.2147/COPD.S179109
3. Doubova SV, Ferreira-Hermosillo A, Perez-Cuevas R, Barsoe C, Gryzbowski-Gainza E, Valencia JE. Socio-demographic and clinical characteristics of type 1 diabetes patients associated with emergency room visits and hospitalizations in Mexico. BMC Health Serv Res. 2018;18: 602. doi:10.1186/s12913-018-3412-3
4. Eisner MD, Katz PP, Lactao G, Iribarren C. Impact of depressive symptoms on adult asthma outcomes. Ann Allergy Asthma Immunol Off Publ Am Coll Allergy Asthma Immunol. 2005;94: 566–74. doi:10.1016/S1081-1206(10)61135-0
5. Ghanei M, Aslani J, Farahani M, Assari S, Saadat SH. Logistic regression model to predict chronic obstructive pulmonary disease exacerbation. Arch Med Sci. 2007;3: 360–366.
6. Guthrie EA, Dickens C, Blakemore A, Watson J, Chew-Graham C, Lovell K, et al. Depression predicts future emergency hospital admissions in primary care patients with chronic physical illness. J Psychosom Res. 2016;82: 54–61. doi:10.1016/j.jpsychores.2014.10.002
7. Himelhoch S, Weller WE, Wu AW, Anderson GF, Cooper LA. Chronic medical illness, depression, and use of acute medical services among Medicare beneficiaries. Med Care. 2004;42: 512–21.
8. Katon WJ, Young BA, Russo J, Lin EHB, Ciechanowski P, Ludman EJ, et al. Association of Depression With Increased Risk of Severe Hypoglycemic Episodes in Patients With Diabetes. Ann Fam Med. 2013;11: 245–250. doi:10.1370/afm.1501
9. Laurence B, Mncube-Barnes FM, Laurence SS, Woods D, Eiland D. Depression and the Likelihood of Hospital Admission from the Emergency Department among Older Patients with HIV. J Health Care Poor Underserved. 2019;30: 131–142. doi:10.1353/hpu.2019.0012
10. Laurence B, Mould-Millman N-K, Nero KEJ, Salter RO, Sagoo PK. Depression and hospital admission in older patients with head and neck cancer: analysis of a national healthcare database. Gerodontology. 2017;34: 284–287. doi:10.1111/ger.12247
11. Mausbach BT, Irwin SA. Depression and healthcare service utilization in patients with cancer. Psychooncology. 2017;26: 1133–1139. doi:10.1002/pon.4133
12. Moraska AR, Chamberlain AM, Shah ND, Vickers KS, Rummans TA, Dunlay SM, et al. Depression, healthcare utilization, and death in heart failure: a community study. Circ Heart Fail. 2013;6: 387–94. doi:10.1161/CIRCHEARTFAILURE.112.000118
13. Niazi S, Frank RD, Sharma M, Roy V, Ames S, Rummans T, et al. Impact of psychiatric comorbidities on health care utilization and cost of care in multiple myeloma. Blood Adv. 2018;2: 1120–1128. doi:10.1182/bloodadvances.2018016717
14. Pan X, Sambamoorthi U. Health care expenditures associated with depression in adults with cancer. J Community Support Oncol. 2015;13: 240–247. doi:10.12788/jcso.0150
15. Poojary P, Saha A, Chauhan K, Simoes P, Sands BE, Cho J, et al. Predictors of Hospital Readmissions for Ulcerative Colitis in the United States: A National Database Study. Inflamm Bowel Dis. 2017;23: 347–356. doi:10.1097/MIB.0000000000001041
16. Ricketts T, Wood E, Soady J, Saxon D, Hulin J, Ohlsen S, et al. The effect of comorbid depression on the use of unscheduled hospital care by people with a long-term condition: A retrospective observational study. J Affect Disord. 2018;227: 366–371. doi:10.1016/j.jad.2017.10.029
17. Schneider A, Lowe B, Meyer FJ, Biessecker K, Joos S, Szecsenyi J. Depression and panic disorder as predictors of health outcomes for patients with asthma in primary care. Respir Med. 2008;102: 359–66. doi:10.1016/j.rmed.2007.10.016
18. Shah R, Haydek C, Mulki R, Qayed E. Incidence and predictors of 30-day readmissions in patients hospitalized with chronic pancreatitis: A nationwide analysis. Pancreatol Off J Int Assoc Pancreatol IAP Al. 2018;18: 386–393. doi:10.1016/j.pan.2018.04.006
19. Sokoreli I, Pauws SC, Steyerberg EW, de Vries G-J, Riistama JM, Tesanovic A, et al. Prognostic value of psychosocial factors for first and recurrent hospitalizations and mortality in heart failure patients: insights from the OPERA-HF study. Eur J Heart Fail. 2018;20: 689–696. doi:10.1002/ejhf.1112
20. Xu W, Collet J-P, Shapiro S, Lin Y, Yang T, Platt RW, et al. Independent effect of depression and anxiety on chronic obstructive pulmonary disease exacerbations and hospitalizations. Am J Respir Crit Care Med. 2008;178: 913–920. doi:10.1164/rccm.200804-619OC

| Questions for the Tool for Quality Assessment of Observational Cohort and Cross-Sectional Studies   1. Was the research question or objective in this paper clearly stated? 2. Was the study population clearly specified and defined? 3. Was the participation rate of eligible persons at least 50%? 4. Were all the subjects selected or recruited from the same or similar populations (including the same time period)? Were inclusion and exclusion criteria for being in the study prespecified and applied uniformly to all participants? 5. Was a sample size justification, power description, or variance and effect estimates provided? 6. For the analyses in this paper, were the exposure(s) of interest measured prior to the outcome(s) being measured? 7. Was the timeframe sufficient so that one could reasonably expect to see an association between exposure and outcome if it existed? 8. For exposures that can vary in amount or level, did the study examine different levels of the exposure as related to the outcome (e.g., categories of exposure, or exposure measured as continuous variable)? 9. Were the exposure measures (independent variables) clearly defined, valid, reliable, and implemented consistently across all study participants? 10. Was the exposure(s) assessed more than once over time? 11. Were the outcome measures (dependent variables) clearly defined, valid, reliable, and implemented consistently across all study participants? 12. Were the outcome assessors blinded to the exposure status of participants? 13. Was loss to follow-up after baseline 20% or less? 14. Were key potential confounding variables measured and adjusted statistically for their impact on the relationship between exposure(s) and outcome(s)? |
| --- |
